# Slow Uptake of PrEP: Behavioral Predictors and the Influence of Price on PrEP Uptake Among MSM with a High Interest in PrEP

**DOI:** 10.1007/s10461-021-03200-4

**Published:** 2021-02-21

**Authors:** Mart van Dijk, John B. F. de Wit, Thomas E. Guadamuz, Joel E. Martinez, Kai J. Jonas

**Affiliations:** 1 Department of Work & Social Psychology, Faculty of Psychology and Neuroscience, Maastricht University, PO Box 616, 6200 MD Maastricht, The Netherlands; 2 Department of Interdisciplinary Social Science, Utrecht University, Utrecht, The Netherlands; 3 Department of Society and Health, Mahidol University, Bangkok, Thailand; 4 Department of Psychology, Princeton University, Princeton, NJ USA

**Keywords:** HIV prevention, MSM, PrEP, Costs

## Abstract

Despite the improved availability and affordability of PrEP in the Netherlands, PrEP uptake is low among men who have sex with men (MSM). To optimize uptake, it is important to identify facilitators and barriers of PrEP use. During our study period, the price of PrEP dropped significantly after generic PrEP was introduced. We investigated whether the price drop predicts PrEP uptake, alongside behavioral and demographic characteristics. Participants (N = 349) were recruited online and completed three questionnaires over a period of 6 months, between February 2017 and March 2019. After 6 months, 159 (45.6%) participants were using PrEP. PrEP uptake was greater among MSM who ever had postexposure prophylaxis (PEP) treatment, among MSM with a better perceived financial situation, and when the price of PrEP dropped. MSM in a tighter perceived financial situation may use PrEP more when it would be free or fully reimbursed.

**Supplementary Information:**

The online version contains supplementary material available at 10.1007/s10461-021-03200-4.

## Resumen

A pesar de la disponibilidad y asequibilidad a PrEP en Los Paises Bajos, el consumo de PrEP es bajo entre hombres que tienen sexo con hombres (HSH). Para optimizar su consumo, es importante identificar los factores facilitadores y las barreras del uso de PrEP. Durante nuestro estudio, el precio de PrEP se redujo significativamente, después de que se introdujo la PrEP genérica al mercado. Investigamos las características demograficas y de conducta y si la caída en el precio predice el consumo de PrEP. Participantes (N = 349) fueron reclutados en linea y completaron tres cuestionarios en un periodo de seis meses, entre Febrero de 2017 y Marzo de 2019. Despues de seis meses, 159 participantes (45.6%) estaban usando PrEP. El consumo de PrEP fue mayor cuando el precio de PrEP bajó y entre HSH que se sometieron a un tratamiento de profilaxis posexposición (PEP) y que tenían una buena situación financiera. HSH con una percibida situacion finaciera más precaria podrían usar PrEP más cuando sea gratis o reembolsada totalmente.

## Introduction

Pre-Exposure Prophylaxis (PrEP) has been found to be an effective biomedical intervention for HIV prevention [1–4]. The World Health Organization (WHO) recommends PrEP for people who are at substantial risk of HIV infection, for example men who have sex with men (MSM) [5]. In the past years, efforts have been made to make PrEP accessible, for example by setting up national PrEP implementation guidelines [6, 7]. Despite its effectiveness and these efforts, the accessibility of PrEP varies greatly per country [8], the uptake of PrEP has been low [9], and the full potential of PrEP at population level has not been reached yet [10]. In the Netherlands, it is estimated that there are currently 3500 individuals on PrEP [8], of which 95% are MSM [11], while it is estimated that 10,000 MSM meet eligibility criteria for PrEP [12, 13]. To increase uptake, it is therefore important to identify facilitators and barriers of PrEP use. While earlier studies investigated behavioral and psychological factors of PrEP uptake, such as sexual risk behaviors, perceived HIV risk, and stigma [14–17], the influence of actual price changes on PrEP use has not been fully investigated, especially not in contexts where universal health care coverage of PrEP is not (yet) available.

Previous studies found several behavioral factors to be related to PrEP use. Compared to MSM who were not using PrEP, PrEP users were more likely to have had a recent STI diagnosis, have used post-exposure prophylaxis (PEP) before, have had condomless anal intercourse, have used recreational drugs or practiced chemsex (i.e., use of certain stimulants in the context of sex), have had sex with HIV positive sex partners, and have a greater number of sex partners [18–21]. In terms of demographic characteristics, PrEP users were found to be of middle age and to have a higher income [18, 20]. While it is reassuring to see that MSM at higher risk of HIV are more likely to be interested in PrEP and to use PrEP, overall PrEP remains “underused”, and some population strata at higher risk are less likely to use PrEP [22–24].

The slow uptake of PrEP so far has been explained by structural and psychosocial barriers, including lack of access to PrEP, doubts about effectiveness, concerns about side-effects, and expected stigma [25–29]. Moreover, the costs of PrEP have been noted as one of the main barriers for PrEP uptake in cross-sectional analysis [30–37]. Notably, in a report drawing on data from 32 European and Asian countries, the price of PrEP was the most common barrier for PrEP uptake [38]. With prices of around € 500 per month for patented Tenofovir-Emtricitabine formulations in Europe and $1600 per month in the U.S., branded PrEP is likely unaffordable for most people in many countries.

In recent years, PrEP has however become more affordable and accessible as a result of the introduction of generic formulations of PrEP and the inclusion of PrEP in health care packages or insurance coverage [8, 39]. PrEP uptake may increase as it becomes more affordable, and this hypothesis is further supported by the finding that uninsured MSM are less likely to use PrEP [23, 40], if PrEP is included in health insurance coverage. Yet, even though the affordability of PrEP is increasing, current pricing may still be a barrier for certain individuals and groups, notably MSM with lower incomes.

In the current study we examine whether the costs of PrEP indeed predict PrEP uptake, alongside behavioral and demographic characteristics. As of 1 January 2018 the price of PrEP in the Netherlands decreased from € 500, to € 50, per month, as a result of the introduction of generic formulations of PrEP [39]. This introduction of generic PrEP allowed us to look more closely into the effects of price on the uptake of PrEP. At the time of our study, PrEP was not included in reimbursement schemes of the national health insurance. The primary way of obtaining PrEP was to buy PrEP at the pharmacy on prescription from the general practitioner [41]. Formal PrEP services, offering PrEP in a co-payment scheme, were implemented in the public health centers as of July 2019, after data collection of our study was finished [11, 42].

## Methods

### Participants and Procedure

Participants were recruited via PrEPnu.nl, the website of the Dutch PrEP advocacy group PrEPnu (Dutch for PrEPnow), between February 2017 and March 2019. Every consenting participant in the baseline survey (T0) received follow-up questionnaires via email after 3 (T1) and 6 months (T2). Participants who did not complete the T1 questionnaire were still encouraged to complete the T2 questionnaire. Participants younger than 18 years old or living with HIV were excluded from participation. All participants who completed at least two questionnaires (T0 + T1/T2) could enter into a raffle to win a € 100,- gift card. The Ethics Review Committee Psychology and Neuroscience of Maastricht University approved this study (ERCPN-174_10_12_2016). In the current study, we did not use the data from the T1 questionnaire, as this resulted in a smaller sample size because some T0 and T2 participants did not complete the T1 questionnaire.

In total, 767 participants completed the baseline (T0) questionnaire. For the current analysis, we only included MSM who were not using PrEP at baseline, and completed the items in the T2 questionnaire that are pertinent for the current analyses. This resulted in a sample size of N = 349. A full description of PrEP use in the sample at all time points is provided in the online supplementary material A.

### Measures

Given the lack of published or validated instruments at the onset of the study, questionnaire items were drawn from the earlier Flash PrEP in Europe study [43], or newly designed by the researchers. Questionnaires were administered online using Qualtrics.com; participants could not revert back to previous questions. The questionnaire was offered in Dutch and English. The full questionnaire can be found on https://osf.io/dm79v/. Below we describe the relevant variables for the current analyses.

#### Sociodemographic Characteristics

In the first questionnaire (T0) participants were asked to indicate their gender, age, relationship status, educational level, financial situation, country of birth, and country of residence. Gender was determined using two questions: gender assigned at birth and current gender. Educational level was indicated by five levels, ranging from no tertiary education to PhD degree. Financial situation was assessed with a 6-point scale: (1) ‘you can’t make ends meet without borrowing’, (2) ‘you are having problems making ends meet’, (3) ‘you are getting by but have to be careful’, (4) ‘things are all right’, (5) ‘you are doing rather well’, and (6) ‘you are doing really well’. The sociodemographic items were not repeated at T1 or T2, as we considered these characteristics to be stable over a period of 6 months. Relationship status was again asked at T2, as this is more likely to change over time.

#### PrEP Related Items

At T2, we asked whether participants were taking PrEP, using a question with sex response options (Yes, I use PrEP daily/Yes, I use PrEP intermittently (more or less every time I have sex)/Yes, I use PrEP recreationally (on demand; during special phases/moments when I have sex)/No, but I have used PrEP before (less than 6 months ago)/No, but I have PrEP used before (more than 6 months ago)/No, I haven’t used PrEP at all). We decided to distinguish between “intermittent” and “recreational” PrEP to capture a possible difference between MSM who use PrEP more frequently (but not daily) and others who use PrEP less systematically and more recreationally or season-based [44–46]. Since the development of our questionnaire (2017) there have been changes in terminology. Currently the common term for “intermittent PrEP” is “on demand PrEP”. There is no consensus regarding the terminology to refer to more recreational or seasonal PrEP use, although it is noted “recreational” use may inadvertently imply non-prescribed PrEP use [47]. Participants who were using PrEP were asked to indicate how they obtained PrEP, with seven response options: via HIV positive friend(s), through PEP treatment, through a PrEP research trial, at a local pharmacy, via a buyers club, at pharmacies abroad, and at online pharmacies.

#### Sexual Risk Behavior

Participants were asked whether they used a condom the last time they had anal intercourse (yes/no). In addition, we asked whether participants had used drugs in a sexual context (yes/no). Participants were also asked to indicate the number of sex partners they had in the past 6 months.

#### Sexual Health

Participants were asked whether they ever had PEP treatment (yes/no) and whether they ever had an STI (yes, in the past 12 months/yes, more than 12 months ago/no).

#### PrEP Pricing

Price of PrEP was dummy coded as “0”, indicating that when the participant completed the T2 questionnaire only branded PrEP was available at pharmacies in the Netherlands at a cost of € 500 (until 01-01-2018), or “1”, indicating that when the participant completed the T2 questionnaire generic PrEP was available at pharmacies in the Netherlands, at a price of €50 or lower (after 01-01-2018).

### Data Analysis

We analyzed the data using IBM SPSS Statistics version 26. We controlled for duplicate participation by participant identifiers. We used descriptive statistics to describe the sociodemographic characteristics of the sample. We analyzed only the data of participants who were not using PrEP at T0 (*N* = 344), to investigate the factors at baseline (T0) potentially related to PrEP initiation after 6 months (T2), using multivariate logistic regression analysis. The following independent variables were included in the model: age, number of sex partners in the past 6 months, educational level, perceived financial situation, relationship status, STI history, having used a condom at last anal intercourse, having used drugs in a sexual context, and having ever had PEP treatment. Additionally, we added the variable ‘price of PrEP’ (at T2) to the model to investigate the influence of the price of PrEP on PrEP initiation. We conducted a post-hoc interaction analysis of the effect of the price of PrEP on PrEP initiation, stratified by different levels of the perceived financial situation [48, 49].

## Results

### Participant Characteristics

Descriptive characteristics of the sample are shown in *Table **1*. Most participants identified their gender as male (344; 98.6%), and the remaining five identified as non-binary or preferred not to answer. The average age of participants was 41 years (range 18–75). About half of participants were single (196; 56.2%), 136 (39.0%) were in an open relationship, and 17 (4.9%) were in a monogamous relationship. More than half of the participants had a Bachelor degree or higher (218; 62.4%), and on average they perceived their financial situation as quite favorable (*M* = 4.35, *SD* = 1.13, median = 4).

**Table 1:** Participant characteristics

	Total sample *N* = 349	PrEP users *N* = 159	Non-PrEP users *N* = 190
Age (years; mean, range)	41 (18–75)	42 (20–66)	40 (18–75)
Gender			
Male	344 (98.6%)	157 (98.7%)	187 (98.4%)
Non-binary/unknown	5 (1.4%)	2 (1.3%)	3 (1.6%)
Born in the Netherlands	281 (80.5%)	125 (78.6%)	156 (82.1%)
Living in the Netherlands	335 (96.0%)	154 (96.9%)	181 (95.3%)
Perceived financial situation (mean, SD; scale 1 = You can't make ends meet without borrowing, to 6 = You are doing really well)	4.35 (1.13)	4.62 (1.02)	4.12 (1.16)
Education level			
Master & PhD	101 (28.9%)	49 (30.8%)	52 (27.4%)
Bachelor	117 (33.5%)	48 (30.2%)	69 (36.3%)
High school & Professional qualification	131 (37.5%)	62 (39.0%)	69 (36.3%)
Relationship status			
Single	196 (56.2%)	86 (54.1%)	110 (57.9%)
In a relationship	17 (4.9%)	5 (3.1%)	12 (6.3%)
In an open relationship	136 (39.0%)	68 (42.8%)	68 (35.8%)
STI			
No	92 (26.4%)	38 (23.9%)	54 (28.4%)
Yes in the past 12 months	116 (33.2%)	65 (40.9%)	51 (26.8%)
Yes more than 12 months ago	141 (40.4%)	56 (35.2%)	85 (44.7%)
Used a condom the last time	161 (46.1%)	76 (47.8%)	85 (44.7%)
Used drugs in a sexual context	174 (49.9%)	85 (53.5%)	89 (46.8%)
Ever had a PEP treatment	41 (11.7%)	24 (15.1%)	17 (8.9%)
Number of sex partners in past 6 months (mean, SD)	14.96 (19.12)	18.26 (24.18)	12.19 (12.95)

Table 2 displays the frequencies of PrEP use at T1 and T2. At T2, 159 (45.6%) participants were using PrEP or had ever used PrEP in the past. Almost half had used PrEP daily (75; 47.2%), 40 used PrEP on demand (25.2%), 32 used PrEP recreationally (20.1%), and 12 had (temporarily) stopped taking PrEP (7.5%). Most participants obtained PrEP via a doctor’s prescription and paid for PrEP themselves at a pharmacy in the Netherlands (104; 65.4%), 31 (19.5%) obtained PrEP from a pharmacy abroad, 10 (6.3%) obtained PrEP through participation in a research trial, 9 (5.7%) obtained PrEP via a buyer’s club, 8 (5.0%) obtained PrEP via online pharmacies abroad, 6 (3.8%) obtained PrEP via PEP treatment, and 3 (1.9%) obtained PrEP via HIV-positive friends. Some participants obtained PrEP via multiple channels. When stratified by financial situation, we found that participants who perceived their financial situation as “really well” were more likely to buy PrEP from pharmacies abroad compared to participants with a lower perceived financial situation (see *Table 6* in *Online supplementary material B*). Participants who perceived their financial situation as “having to be careful about expenses” or lower were less likely to use PrEP at all.

**Table 2:** Frequencies of PrEP use at T2 stratified by PrEP use at T1

	PrEP use at T2						
	Daily	Intermittent	Recreationally	Used PrEP less than 6 months ago	Used PrEP more than 6 months ago	Did not use PrEP at all	Total
PrEP use at T1							
Daily	47	5	4	4	0	0	60
Intermittent	3	17	6	0	0	0	26
Recreationally	0	5	6	1	0	0	12
Used PrEP less than 6 months ago	1	1	0	1	0	0	3
Used PrEP more than 6 months ago	0	0	0	0	2	0	2
Did not use PrEP at all	18	7	13	2	1	162	203
Missing	6	5	3	0	1	28	43
Total	75	40	32	8	4	190	349

The participants in this selection were not using PrEP at baseline (T0)

### Predictors of PrEP Initiation After 6 Months

The outcomes of the logistic regression analysis of correlates of PrEP initiation are shown in *Table **3*. Significant multivariable correlates of PrEP initiation included perceived financial situation (aOR 1.50, 95% CI 1.21–1.87), having ever had PEP treatment (aOR 2.34, 95% CI 1.12–4.86), and the price of PrEP (aOR 1.91, 95% CI 1.09–3.32).

**Table 3:** Bivariate and multivariate logistic regression examining correlates of PrEP initiation

	Bivariate		Multivariate	
	OR	95% CI OR	aOR	95% CI aOR
Age	1.01	1.00–1.03	1.01	0.99–1.03
Number of sex partners in past 6 months	1.02**	1.01–1.04	1.01	1.00–1.03
Perceived financial situation	1.52***	1.24–1.86	1.50***	1.21–1.87
Education level				
Master & PhD	Ref.		Ref.	
Bachelor	0.74	0.43–1.26	0.97	0.54–1.74
High school & Professional qualification	0.95	0.57–1.60	1.11	0.63–1.96
Relationship status				
Single	Ref		Ref	
In an open relationship	1.28	0.83–1.98	1.15	0.70–1.87
In a relationship	0.53	0.18–1.57	0.46	0.14–1.51
STI				
Never had an STI	Ref.		Ref.	
Had an STI in the past 12 months	1.81*	1.04–3.15	1.57	0.83–2.95
Had an STI more than 12 months ago	0.94	0.55–1.60	0.72	0.40–1.31
Not used a condom the last time^a^	1.13	0.74–1.73	1.17	0.72–1.88
Used drugs in a sexual context^a^	1.30	0.86–1.99	1.26	0.78–2.06
Ever had a PEP treatment^a^	1.81	0.93–3.50	2.34*	1.12–4.86
Price of PrEP^b^	1.71*	1.03–2.84	1.91*	1.09–3.32

^a^The reference category for these variables is “no”

^b^The variable ‘Price of PrEP’ was coded with 0 (when the participant completed the survey at the time when PrEP was € 500 per month, before 01-01-2018) and 1 (when the participant completed the survey at the time when PrEP was € 50 per month, after 01-01-2018)

*p < 0.05

**p < 0.01

***p < 0.001

χ^2^ (13, *N* = 349) = 46.08, *p* < 0.001, Nagelkerke *R*^2^ = 0.165

In a post-hoc analysis we investigated the effect of the price of PrEP on PrEP initiation at three levels of perceived financial situation: The average level of perceived financial situation in the sample and at levels of perceived financial situation one standard deviation below as well as one standard deviation above the average perceived financial situation (Table 4). We found that the price of PrEP was only related to PrEP initiation when perceived financial situation was at an average level (aOR 1.83, 95% CI 1.06–3.17).

**Table 4:** Examining the conditional effect of price of PrEP on PrEP use at different levels of perceived financial situation in the multivariate logistic regression

	aOR	95% CI aOR
One SD below mean financial situation (3.22)	2.06	0.88–4.82
At mean financial situation (4.35)	1.83*	1.06–3.17
One SD above mean financial situation (5.47)	1.62	0.75–3.50

*p < 0.05

## Discussion

The aim of this study was to investigate what sociodemographic and behavioral factors predict PrEP uptake. We in particular investigated whether the price decrease of PrEP, from € 500,- to € 50,- per month, resulting from the introduction of generic formulations of PrEP, is associated with an increased PrEP uptake. This study found that a better perceived financial situation, having ever had PEP treatment, and the price decrease of PrEP were significantly related to PrEP initiation. This is in line with our hypotheses and findings of earlier studies. However, while earlier cross-sectional studies reported the price of PrEP to be an important overall barrier for intended PrEP uptake [30–38], we found more specifically that the price decrease of PrEP was only related to an increase in PrEP uptake among participants with an average perceived financial situation. This might indicate that the current price reduction of PrEP (from € 500 to € 50 per month for the Dutch context) did not impact MSM in more unfavorable nor more favorable financial situations, likely for different reasons. MSM in a favorable perceived financial situation may use PrEP anyway, regardless of price level, because the use of PrEP does not have a substantial impact on their financial situation. On the other hand, MSM in an unfavorable perceived financial situation may find the price of € 50 per month still too high and may be not be able to afford PrEP at this price. This indicates a need for the inclusion PrEP in health insurance or the implementation reimbursement schemes to increase PrEP uptake among less affluent MSM [23, 40].

While having ever had PEP treatment was a significant predictor of PrEP initiation, other variables related to sexual risk behavior, such as number of sex partners in the past 6 months, history of STIs, condom use, and substance use were not significantly related to PrEP initiation after 6 months. These variables are considered key indicators for PrEP use and eligibility for PrEP, as they reflect an increased risk of HIV, and we therefore expected these to be significant predictors of PrEP initiation. A possible explanation for the lack of such a relationship is that participants in our sample overall had a high prevalence of sexual risk behaviors; most would be eligible for PrEP. Hence, their sexual risk behaviors are unlikely to distinguish between those who start taking PrEP or not (i.e., ceiling effect). This is in line with an earlier study that found that despite being an appropriate candidate for PrEP, and contemplating PrEP use, MSM do not always initiate PrEP use [9]. It was argued that PrEP initiation could be increased if PrEP providers apply motivational interviewing techniques to help MSM decide on PrEP use. It is important to recognize the role of healthcare providers in PrEP initiation. In our study we found that having had PEP treatment is related to PrEP use. This might be a result of the Dutch national guideline that instructs health care providers to encourage MSM to continue PrEP use directly after a PEP treatment [6]. This indicates that health care providers should be trained to recognize eligible candidates for PrEP and to be confident to prescribe PrEP to them.

Notably, only 45.6% of the participants in our study were using PrEP after 6 months follow-up. In this sample, we expected a higher PrEP uptake because of the high interest in PrEP among the participants. In the context of the Transtheoretical Model of Change, as applied to the PrEP cascade by Parsons et al. [9], most participants in our study were either in the stage of PrEP contemplation (i.e. willing to take PrEP) or the stage of PrEParation (i.e. intending to take PrEP), because the participants were recruited on a website where they could find detailed information on how to obtain PrEP. Thus, interest and knowledge would not be limiting factors to procure PrEP for the participants in this sample. MSM moved from the PrEParation stage to the action stage as soon as the price of PrEP dropped. Also other studies found large gaps between interest in PrEP and PrEP uptake [9, 50, 51]. It seems that structural barriers play a larger role in explaining this gap compared to psychosocial and behavioral factors. For example, among young Latino MSM it was found that structural syndemic factors, such as poverty and unstable housing, limit PrEP uptake despite high interest in PrEP [50]. In our study we found that the perceived financial situation and the price of PrEP were the most important factors in predicting PrEP uptake.

There are a few limitations to this study. We recruited participants using convenience sampling, limiting the representativeness of this study for the whole MSM population. The sample consisted mostly of highly educated MSM who were born in the Netherlands. MSM with lower education levels and migrant MSM may face other challenges when accessing PrEP. It is important to study the specific needs of these MSM subgroups, in particular because non-Western migrant MSM in the Netherlands have an increased risk of acquiring HIV [52]. Still, our study highlights that even among non-minority MSM in the Netherlands the price of PrEP and their financial situation are significant factors determining access to PrEP. The findings relate specifically to MSM with a high interest in PrEP, and are therefore mostly relevant for explaining the gap between a high interest in PrEP and a low uptake of PrEP [38]. Another limitation of this study is related to the assessment of the price of PrEP. We used the price of PrEP in pharmacies in the Netherlands to construct a variable for the price of PrEP that could be included in the regression analysis. However, some participants obtained PrEP informally (e.g., via pharmacies abroad), so the price of PrEP in pharmacies in the Netherlands may not have influenced their PrEP initiation. Monitoring of prices for PrEP at the time of the study in online pharmacies and through health care providers showed prices quite similar to the reduced price in the Netherlands, with a lower bound of € 30,- in Thailand and average prices around € 50,-. Another limitation is that the variable “price of PrEP” may not merely reflect the change in the price of PrEP, but may also reflect time effects. We did an additional analysis (see *online supplementary material C*) to control for possible time effects, and found no evidence that participants were more likely to use PrEP later in time. This indicates that the effect of the variable “price of PrEP” indeed captures an effect of the price drop of PrEP.

A strength of this study is that it is the first to collect data over a time span in which the price of PrEP significantly changed, allowing us to investigate the relationship between an actual, real-world change in the price of PrEP and PrEP uptake. These findings are not only relevant for the Netherlands, but also for other countries. In 2019, PrEP was not included in reimbursement schemes in 37 (out of 53 reporting) countries in Europe, underscoring that costs of PrEP likely continue to impact PrEP use [53]. We further expect that the results of our study remain relevant in the future, even when the price of branded PrEP may be (further) lowered, as new types of formulation (e.g., Emtricitabine/tenofovir alafenamide; [54]) or administration (e.g., injectables, implants [55]) may result in new pricing barriers. Pricing barriers may also continue to exist after the introduction of generic formulations of PrEP, because generic formulations of PrEP are not always substantially cheaper than branded Truvada [56].

To optimize PrEP uptake among MSM with a high interest in PrEP with limited financial resources, the cost of PrEP play an important role. The introduction of lower price generic formulations of PrEP led to an increase in PrEP uptake in the Netherlands. However, PrEP continued to be used by MSM in a favorable perceived financial situation. MSM in an unfavorable perceived financial situation may be more likely to use PrEP if it is available free of charge, through health insurance, or fully reimbursed, through a government scheme.

## Supplementary Information

Below is the link to the electronic supplementary material.

Supplementary file1 (DOCX 52 KB)

## References

[1] Fonner VA, Dalglish SL, Kennedy CE, Baggaley R, O’Reilly KR, Koechlin FM (2016). Effectiveness and safety of oral HIV preexposure prophylaxis for all populations. AIDS.

[2] Grant RM, Lama JR, Anderson PL, McMahan V, Liu AY, Vargas L (2010). Preexposure chemoprophylaxis for HIV prevention in men who have sex with men. N Engl J Med.

[3] Grant RM, Anderson PL, McMahan V, Liu A, Amico KR, Mehrotra M (2014). Uptake of pre-exposure prophylaxis, sexual practices, and HIV incidence in men and transgender women who have sex with men: a cohort study. Lancet Infect Dis.

[4] Molina JM, Charreau I, Spire B, Cotte L, Chas J, Capitant C (2017). Efficacy, safety, and effect on sexual behaviour of on-demand pre-exposure prophylaxis for HIV in men who have sex with men: an observational cohort study. Lancet HIV.

[5] World Health Organization (2015). Guideline on when to start antiretroviral therapy and on pre-exposure prophylaxis for HIV.

[6] Hoornenborg E, Rijnders B. HIV Pre-expositie profylaxe (PrEP) richtlijn Nederland. 2016. http://richtlijnhiv.nvhb.nl/images/2/22/PrEP-richtlijn-Nederland-8-september-2016-met-logos.pdf

[7] CDC. Preexposure prophylaxis for the prevention of HIV infection in the United States - 2017 update: a clinical practice guideline. [Internet]. Preexposure prophylaxis for the prevention of HIV infection in the United States - 2017 update: a clinical practice guideline. 2017. https://stacks.cdc.gov/view/cdc/53509

[8] AVAC. PrEP Watch. 2020. https://www.prepwatch.org/

[9] Parsons JT, Rendina HJ, Lassiter JM, Whitfield THF, Starks TJ, Grov C (2017). Uptake of HIV pre-exposure prophylaxis (PrEP) in a national cohort of gay and bisexual men in the United States: the Motivational PrEP Cascade HHS Public Access. J Acquir Immune Defic Syndr.

[10] Pyra MN, Haberer JE, Hasen N, Reed J, Mugo NR, Baeten JM (2019). Global implementation of PrEP for HIV prevention: setting expectations for impact. J Int AIDS Soc.

[11] Staritsky LE, Van Aar F, Visser M, Op de Coul ELM, Heijne J, Götz HM, et al. Sexually Transmitted Infections in the Netherlands in 2019. RIVM report 2020–0052. 2020.

[12] Hoornenborg E, Krakower DS, Prins M, Mayer KH (2017). Pre-exposure prophylaxis for MSM and transgender persons in early adopting countries. AIDS.

[13] Reitsema M, van Hoek AJ, van der Loeff MS, Hoornenborg E, van Sighem A, Wallinga J (2020). Preexposure prophylaxis for men who have sex with men in the Netherlands. AIDS.

[14] Blumenthal J, Jain S, Mulvihill E, Sun S, Hanashiro M, Ellorin E (2019). Perceived versus calculated HIV risk: implications for pre-exposure prophylaxis uptake in a randomized trial of men who have sex with men. J Acquir Immune Defic Syndr.

[15] Golub SA, Fikslin RA, Goldberg MH, Peña SM, Radix A (2019). Predictors of PrEP uptake among patients with equivalent access. AIDS Behav.

[16] Dubov A, Altice FL, Fraenkel L (2018). An information–motivation–behavioral skills model of PrEP uptake. AIDS Behav.

[17] Haire B (2015). Preexposure prophylaxis-related stigma: strategies to improve uptake and adherence &ndash; a narrative review. HIV/AIDS Res Palliat Care.

[18] Bourne A, Alba B, Garner A, Spiteri G, Pharris A, Noori T (2019). Use of, and likelihood of using, HIV pre-exposure prophylaxis among men who have sex with men in Europe and Central Asia: Findings from a 2017 large geosocial networking application survey. Sex Transm Infect.

[19] Eaton LA, Matthews DD, Bukowski LA, Friedman MR, Chandler CJ, Whitfield DL (2018). Elevated HIV prevalence and correlates of PrEP use among a community sample of black men who have sex with men. J Acquir Immune Defic Syndr.

[20] Holloway IW, Dougherty R, Gildner J, Beougher SC, Pulsipher C, Montoya JA (2017). Brief report: PrEP uptake, adherence, and discontinuation among California YMSM using geosocial networking applications. J Acquir Immune Defic Syndr.

[21] Okafor CN, Gorbach PM, Ragsdale A, Quinn B, Shoptaw S (2017). Correlates of preexposure prophylaxis (PrEP) use among men who have sex with men (MSM) in Los Angeles, California. J Urban Heal.

[22] Shover CL, Javanbakht M, Shoptaw S, Bolan RK, Lee SJ, Parsons JT (2018). HIV preexposure prophylaxis initiation at a large community clinic: differences between eligibility, awareness, and uptake. Am J Public Heal.

[23] Kuhns LM, Hotton AL, Schneider J, Garofalo R, Fujimoto K (2017). Use of pre-exposure prophylaxis (PrEP) in young men who have sex with men is associated with race, sexual risk behavior and peer network size. AIDS Behav.

[24] Marcus JL, Hurley LB, Hare CB, Silverberg MJ, Volk JE (2016). Disparities in uptake of HIV preexposure prophylaxis in a large integrated health care system. Am J Public Health.

[25] Hannaford A, Lipshie-Williams M, Starrels JL, Arnsten JH, Rizzuto J, Cohen P (2018). The use of online posts to identify barriers to and facilitators of HIV pre-exposure prophylaxis (PrEP) among men who have sex with men: a comparison to a systematic review of the peer-reviewed literature. AIDS Behav.

[26] Calabrese SK, Underhill K (2015). How stigma surrounding the use of HIV preexposure prophylaxis undermines prevention and pleasure: a call to destigmatize “Truvada Whores”. Am J Public Health.

[27] Thomann M, Grosso A, Zapata R, Chiasson MA (2018). ‘WTF is PrEP?’: attitudes towards pre-exposure prophylaxis among men who have sex with men and transgender women in New York City. Cult Health Sex.

[28] Golub SA, Gamarel KE, Rendina HJ, Surace A, Lelutiu-Weinberger CL (2013). From efficacy to effectiveness: facilitators and barriers to PrEP acceptability and motivations for adherence among MSM and transgender women in New York City. AIDS Pat Care STDS.

[29] Pinto RM, Berringer KR, Melendez R, Mmeje O (2018). Improving PrEP implementation through multilevel interventions: a synthesis of the literature. AIDS Behav.

[30] Arnold T, Brinkley-Rubinstein L, Chan PA, Perez-Brumer A, Bologna ES, Beauchamps L (2017). Social, structural, behavioral and clinical factors influencing retention in Pre-Exposure Prophylaxis (PrEP) care in Mississippi. PLoS ONE.

[31] Dubov A, Ogunbajo A, Altice FL, Fraenkel L (2019). Optimizing access to PrEP based on MSM preferences: results of a discrete choice experiment. AIDS Care.

[32] Bauermeister J, Meanley S, Pingel E, Soler J, Harper G (2014). PrEP awareness and perceived barriers among single young men who have sex with men. Curr HIV Res.

[33] Goparaju L, Praschan NC, Jeanpiere LW, Experton LS, Young MA, Kassaye S (2017). Stigma, partners, providers and costs: potential barriers to PrEP uptake among US women. J AIDS Clin Res..

[34] Rice WS, Stringer KL, Sohail M, Crockett KB, Atkins GC, Kudroff K (2019). Accessing pre-exposure prophylaxis (PrEP): perceptions of current and potential PrEP users in Birmingham, Alabama. AIDS Behav.

[35] Pérez-Figueroa RE, Kapadia F, Barton SC, Eddy JA, Halkitis PN (2015). Acceptability of prep uptake among racially/ethnically diverse young men who have sex with men: the p18 study. AIDS Educ Prev.

[36] Kubicek K, Arauz-Cuadra C, Kipke MD (2015). Attitudes and perceptions of biomedical HIV prevention methods: voices from young men who have sex with men. Arch Sex Behav.

[37] Schwartz J, Grimm J (2017). PrEP on Twitter: information, barriers, and stigma. Health Commun.

[38] Hayes R, Schmidt AJ, Pharris A, Azad Y, Brown AE, Weatherburn P (2019). Estimating the ‘PrEP Gap’: how implementation and access to PrEP differ between countries in Europe and Central Asia in 2019. Eurosurveillance.

[39] PrEPnu. Generic PrEP available in selected pharmacies for €99,50. 2017. https://www.prepnu.nl/en/2017/10/27/generic-prep-pharmacies/

[40] Patel RR, Mena L, Nunn A, McBride T, Harrison LC, Oldenburg CE (2017). Impact of insurance coverage on utilization of pre-exposure prophylaxis for HIV prevention. PLoS ONE.

[41] Van Bergen J. De rol van de huisarts bij PrEP revisited. 2019. https://www.soaaids.nl/nl/seksoa-magazine/rol-van-huisarts-bij-prep-revisited

[42] Ministry of Health Welfare and Sport. Kamerbrief over start van verstrekking en medische begeleiding van PrEP. 2019. https://www.rijksoverheid.nl/documenten/kamerstukken/2019/06/26/kamerbrief-over-start-van-verstrekking-en-medische-begeleiding-van-prep

[43] AIDES. First Results of the Flash!PrEP in Europe Online Survey. 2016. https://www.aides.org/FlashPrEPinEurope

[44] Hojilla JC, Koester KA, Cohen SE, Buchbinder S, Ladzekpo D, Matheson T (2016). Sexual behavior, risk compensation, and HIV prevention strategies among participants in the San Francisco PrEP demonstration project: a qualitative analysis of counseling notes. AIDS Behav.

[45] Elsesser SA, Oldenburg CE, Biello KB, Mimiaga MJ, Safren SA, Egan JE (2016). Seasons of risk: anticipated behavior on vacation and interest in episodic antiretroviral pre-exposure prophylaxis (PrEP) among a large national sample of US men who have sex with men (MSM). Aids Behav.

[46] Underhill K, Guthrie KM, Colleran C, Calabrese SK, Operario D, Mayer KH (2018). Temporal fluctuations in behavior, perceived HIV risk, and willingness to use pre-exposure prophylaxis (PrEP). Arch Sex Behav.

[47] Kuo C, Giovenco D, DeAtley T, Hoare J, Underhill K, Atujuna M (2020). Recreational use of HIV antiretroviral medication and implications for HIV pre-exposure prophylaxis and treatment. AIDS Behav.

[48] Ai C, Norton EC (2003). Interaction terms in logit and probit models. Econ Lett.

[49] Hayes AF, Matthes J (2009). Computational procedures for probing interactions in OLS and logistic regression: SPSS and SAS implementations. Behav Res Methods.

[50] Blashill AJ, Brady JP, Rooney BM, Rodriguez-Diaz CE, Horvath KJ, Blumenthal J (2020). Syndemics and the PrEP cascade: results from a sample of young Latino men who have sex with men. Arch Sex Behav.

[51] Rolle C-P, Rosenberg ES, Siegler AJ, Sanchez TH, Luisi N, Weiss K (2017). Challenges in translating PrEP interest into uptake in an observational study of young black MSM. J AIDS J Acquir Immune Defic Syndr.

[52] Visser M, Van Aar F, Van Oeffelen AAM, Op de Coul ELM, Hofstraat SHI, Heijne J, et al. Sexually transmitted infections including HIV, in the Netherlands in 2016. 2017. https://www.rivm.nl/publicaties/sexually-transmitted-infections-including-hiv-in-netherlands-in-2016

[53] European Centre for Disease Prevention and Control. Pre-exposure prophylaxis for HIV prevention in Europe and Central Asia. Monitoring implementation of the Dublin Declaration on partnership to fight HIV/AIDS in Europe and Central Asia – 2018/19 progress report. Stockholm; 2019. https://www.ecdc.europa.eu/sites/default/files/documents/HIV-pre-exposure-prophylaxis-evidence-2019_0.pdf

[54] U.S. Food and Drug Administration. FDA approves second drug to prevent HIV infection as part of ongoing efforts to end the HIV epidemic. 2019. https://www.fda.gov/news-events/press-announcements/fda-approves-second-drug-prevent-hiv-infection-part-ongoing-efforts-end-hiv-epidemic

[55] Coelho LE, Torres TS, Veloso VG, Landovitz RJ, Grinsztejn B (2019). Pre-exposure prophylaxis 2.0: new drugs and technologies in the pipeline. Lancet HIV.

[56] Poz.com. First Generic Truvada Now Available in the United States. 2020. https://www.poz.com/article/first-generic-truvada-now-available-united-states

