# Supplementary material for: Slow Uptake of PrEP: Behavioral Predictors and the Influence of Price on PrEP Uptake Among MSM with a High Interest in PrEP
Source: AIDS Behav. 2021 Feb 21;25(8):2382–90. doi: 10.1007/s10461-021-03200-4 (PMC8222036; doi:10.1007/s10461-021-03200-4)
Supplement: Supplementary file 1 — Supplementary file1 (DOCX 52 KB) [file 10461_2021_3200_MOESM1_ESM.docx]

Online supplementary material A

| Table 5: Frequencies of PrEP use at all time points in the full sample (N=767), stratified by PrEP use at baseline T0. | | | | | |
| --- | --- | --- | --- | --- | --- |
|  | T0 |  | T1 |  | T2 |
| PrEP users | 144 | PrEP users | 100 | PrEP users | 92 |
|  |  | Used PrEP before | 6 | Used PrEP before | 3 |
|  |  | Not using PrEP | 1 | Not using PrEP | 1 |
|  |  | Missing | 37 | Missing | 48 |
| Not using PrEP | 613 | PrEP users | 126 | PrEP users | 162 |
|  |  | Used PrEP before | 9 | Used PrEP before | 14 |
|  |  | Not using PrEP | 269 | Not using PrEP | 201 |
|  |  | Missing | 209 | Missing | 236 |
| Missing | 10 | PrEP users | 2 | PrEP users | 1 |
|  |  | Used PrEP before | 0 | Used PrEP before | 0 |
|  |  | Not using PrEP | 2 | Not using PrEP | 2 |
|  |  | Missing | 6 | Missing | 7 |

Please note that this table consists of the raw frequencies, without any filters. Therefore, the frequencies are not the same as reported in our paper. In the analysis in our paper we only used the data of participants who were not using PrEP at T0, and completed the T2 questionnaire, and completed the items at T0 that were used in the regression analysis.

Online supplementary material B

Table 6: Source of PrEP procurement stratified by perceived financial situation. Please note that the total frequency exceeds the number of PrEP users, as participants could report multiple sources of procurement.

| Financial situation | Via HIV-positive friends | Through PEP treatment | Prescription and local pharmacy | Buyers club | Pharmacy abroad | Pharmacy online (abroad) | PrEP research trial | Total |
| --- | --- | --- | --- | --- | --- | --- | --- | --- |
| You can’t make ends meet without borrowing | 0 | 0 | 1 | 0 | 0 | 0 | 0 | 1 |
| You are having problems making ends meet | 1 | 0 | 0 | 0 | 0 | 0 | 0 | 1 |
| You are getting by but have to be careful | 0 | 1 | 10 | 0 | 5 | 0 | 1 | 17 |
| Things are all right | 0 | 4 | 33 | 1 | 8 | 1 | 2 | 49 |
| You are doing rather well | 1 | 1 | 40 | 7 | 8 | 3 | 4 | 64 |
| You are doing really well | 1 | 0 | 20 | 1 | 10 | 4 | 3 | 39 |
| Total | 3 | 6 | 104 | 9 | 31 | 8 | 10 | 171 |

Online supplementary material C

In our analysis, the variable “price of PrEP” reflected the introduction of generic formulations of PrEP, and the related price decrease, in the Netherlands. The variable “price of PrEP” was dummy coded with “0” when the participant completed the T2 questionnaire at the moment when PrEP costed € 500,- at Dutch pharmacies (until 01-01-2018) and with “1” when the participant completed the T2 questionnaire at the moment when PrEP costed € 50,- at Dutch pharmacies (after 01-01-2018). However, it can be argued that this variable may not merely reflect the decrease in the price of PrEP, but instead may also reflect time effects. For example, over time MSM may become more familiar with PrEP, and may therefore initiate PrEP use.

To investigate the effect of time more precisely, we added the variable “time” in the regression model, whereby “time” reflects the month in which the participant completed the T2 survey, coded as “1” for October 2017 up to “24” for September 2019. Since the variables “price of PrEP” and “time” have a high overlap regarding their underlying construct, the correlation between them is too high to include them as independent predictors at the same time in the regression model. Therefore, we conducted two logistic regression analyses including “time” but not “price of PrEP”: The first logistic regression analysis covered the data (*N* = 84) during the period when the price of PrEP was high (€ 500,-). The second logistic regression analysis covered the data (*N* = 265) during the period when the price of PrEP was low (€ 50,-).

*Tables 5 and 6* show the results of these two logistic regression analyses. In both analyses, “time” is not significantly related to PrEP initiation, suggesting that PrEP use does not increase over time because of, for example, becoming familiar with PrEP. These findings strengthen our interpretation that the price decrease of PrEP is related to an increase in PrEP use.

| Table 7: Multivariable logistic regression examining correlates of PrEP initiation during the period when the price of PrEP was high (€ 500,-). | | |
| --- | --- | --- |
|  | aOR | 95% CI aOR |
| Age | 0.98 | 0.93 – 1.04 |
| Number of sex partners in past 6 months | 1.02 | 0.99 – 1.05 |
| Perceived financial situation | 1.51 | 0.91 – 2.52 |
| Education level |  |  |
| Master & PhD |  |  |
| Bachelor | 0.31 | 0.08 – 1.21 |
| High school & Professional qualification | 0.75 | 0.21 – 2.66 |
| Relationship status |  |  |
| Single |  |  |
| In an open relationship | 0.76 | 0.23 – 2.49 |
| In a relationship | 1.34 | 0.15 – 12.28 |
| STI |  |  |
| Never had an STI |  |  |
| Had an STI in the past 12 months | 2.41 | 0.53 – 11.03 |
| Had an STI more than 12 months ago | 0.97 | 0.21 – 4.55 |
| Not used a condom the last time^a^ | 1.30 | 0.39 – 4.28 |
| Used drugs in a sexual context^a^ | 1.10 | 0.34 – 3.49 |
| Ever had a PEP treatment^a^ | 1.44 | 0.29 – 7.27 |
| Time^b^ | 0.70 | 0.36 – 1.35 |
| a The reference category for these variables is “no”. | | |
| b The variable “time” reflects the month in which the participant completed the T2 survey, coded as “1” for October 2017 up to “24” for September 2019. | | |
| χ^2^ (13, *N* = 84) = 16.14, *p* = .24, Nagelkerke *R*^2^ = .240 | | |

| Table 8: Multivariable logistic regression examining correlates of PrEP initiation during the period when the price of PrEP was low (€ 50,-). | | |  |
| --- | --- | --- | --- |
|  | aOR | 95% CI aOR | |
| Age | 1.01 | 0.99 – 1.04 | |
| Number of sex partners in past 6 months | 1.02 | 1.00 – 1.03 | |
| Perceived financial situation | 1.47** | 1.15 – 1.89 | |
| Education level |  |  | |
| Master & PhD |  |  | |
| Bachelor | 1.25 | 0.64 – 2.45 | |
| High school & Professional qualification | 1.27 | 0.66 – 2.44 | |
| Relationship status |  |  | |
| Single |  |  | |
| In an open relationship | 1.36 | 0.77 – 2.39 | |
| In a relationship | 0.32 | 0.07 – 1.39 | |
| STI |  |  | |
| Never had an STI |  |  | |
| Had an STI in the past 12 months | 1.51 | 0.73 – 3.13 | |
| Had an STI more than 12 months ago | 0.64 | 0.33 – 1.25 | |
| Not used a condom the last time^a^ | 1.28 | 0.73 – 2.22 | |
| Used drugs in a sexual context^a^ | 1.47 | 0.83 – 2.61 | |
| Ever had a PEP treatment^a^ | 2.56* | 1.08 – 6.09 | |
| Time^b^ | 1.04 | 0.98 – 1.09 | |
| a The reference category for these variables is “no”. | | |  |
| b The variable “time” reflects the month in which the participant completed the T2 survey, coded as “1” for October 2017 up to “24” for September 2019. | | |  |
| * *p* < .05 | | |  |
| ** *p* < .01 | | |  |
| χ^2^ (13, *N* = 265) = 35.83, *p* < .001, Nagelkerke *R*^2^ = .169 | | |  |
